# Pharmacological interventions in corticobasal degeneration: a review

**DOI:** 10.1590/1980-57642020dn14-030006

**Published:** 2020

**Authors:** Leonardo Caixeta, Victor de Melo Caixeta, Yanley Lucio Nogueira, Tales Alexandre Aversi-Ferreira

**Affiliations:** 1 Department of Neurology, School of Medicine, Universidade Federal de Goiás - Goiânia, GO, Brazil.; 2 Dementia Outpatient Clinic, Hospital das Clínicas, Universidade Federal de Goiás - Goiânia, GO, Brazil.; 3 ASMIGO Hospital, Neuropsychology Research Center - Goiânia, GO, Brazil.; 4 Laboratory of Biomathematics and Physical Anthropology, Department of Structural Biology, Institute of Biomedical Sciences, Universidade Federal de Alfenas - Alfenas, Brazil.

**Keywords:** corticobasal degeneration, corticobasal syndrome, dopaminergic therapy, degeneração corticobasal, síndrome corticobasal, terapia dopaminérgica, tratamento

## Abstract

Corticobasal degeneration (CBD) is a sporadic tauopathy that presents with a varied combination of motor, cognitive, and behavioral features, making its diagnosis difficult. CBD has high morbidity and poor prognosis, with no effective therapy at present. We searched the PubMed/MEDLINE database for articles published from 1990 to 2019, using the keywords “corticobasal degeneration” AND “treatment.” The PRISMA method was adopted. Retrieved articles were characterized as having one of two methodological approaches: (1) studies aimed at primary tauopathy treatment and (2) symptomatic management. Review articles (based on CBD expert groups), case reports, case series, and pilot clinical trials were selected. Few attempts have been made to study drug options and drug efficacy in CBD systematically, and an effective treatment is not yet available. Treatment is symptomatic and based on similarity with other diseases due to the scarcity of studies specifically addressing CBD. CBD seems not to spark interest in more clinical trials for its low prevalence and reliability in clinical diagnosis.

## RESUMO.

A degeneração corticobasal (DCB) é uma tauopatia esporádica que se apresenta com uma combinação variada de características motoras, cognitivas e comportamentais, dificultando seu diagnóstico. O CBD tem alta morbidade e mau prognóstico, sem terapia efetiva no momento. Pesquisamos o banco de dados PubMed / MEDLINE para artigos publicados de 1990 a 2019, usando as palavras-chave "degeneração corticobasal" e "tratamento". O método PRISMA foi adotado. Os artigos recuperados foram caracterizados como tendo uma de duas abordagens metodológicas: (1) estudos voltados para o tratamento da tauopatia primária e (2) manejo sintomático. Artigos de revisão (baseados em grupos de especialistas em CBD), relatos de casos, séries de casos e ensaios clínicos piloto foram selecionados. Poucas tentativas foram feitas para estudar as opções de drogas e eficácia de drogas no CBD de forma sistemática, e um tratamento eficaz ainda não está disponível. O tratamento é sintomático e baseado na semelhança com outras doenças devido à escassez de estudos que abordem especificamente o CBD. O CBD parece não despertar o interesse em mais ensaios clínicos por sua baixa prevalência e confiabilidade no diagnóstico clínico.

## INTRODUCTION

Corticobasal degeneration (CBD) is a neurodegenerative condition caused by a progressive pathological accumulation of tau protein in neurons and glia.^1^ The clinical phenotype of CDB is characterized by a varied combination of motor, cognitive, and behavioral features, usually confused with Alzheimer’s disease (AD) or Parkinson’s disease.^2^ Its classical clinical presentation consists of asymmetric parkinsonism and cortical signals, such as apraxia, cortical sensory loss, and alien limb syndrome. However, possible clinical phenotypes are highly variable, including, besides corticobasal syndrome (CBS), a frontal-behavioral syndrome with spatial changes, progressive aphasic syndrome, progressive supranuclear syndrome similar to progressive supranuclear palsy (PSP-like), and a predominant cognitive phenotype frequently mistaken for AD.^1^ ^,^ ^2^ Recent research revealed that CBD could be caused by different pathological conditions, often resulting in erroneous diagnoses.^3^

CBD presents high morbidity and poor prognosis because of the lack of effective therapy at present. Treatment is symptomatic and based on features of other similar diseases due to the scarcity of studies focused on CBD.^4^ ^,^ ^5^ ^,^ ^6^ ^,^ ^7^

In this review, we will discuss specific and symptomatic treatments for motor and non-motor, cognitive, and behavioral symptoms currently used for CBD, based on a critical analysis of developments in this area.

## METHODS

The process to select the articles for analysis followed the Preferred Reporting Items for Systematic Reviews and Meta-Analyses (PRISMA) guidelines.

We searched the PubMed database for articles published from January 1990 to December 2019 with the keywords “corticobasal degeneration” AND “corticobasal treatment,” finding a total of the 238 papers for both keywords, considering only the full texts.

From the Scopus/Capes database, we retrieved 87 studies with corticobasal degeneration in the title and corticobasal treatment as the subject. The search for corticobasal treatment in the title and corticobasal degeneration as the subject resulted in 14 articles. When considering both terms as keywords, 12 papers were retrieved.

**Figure 1.**
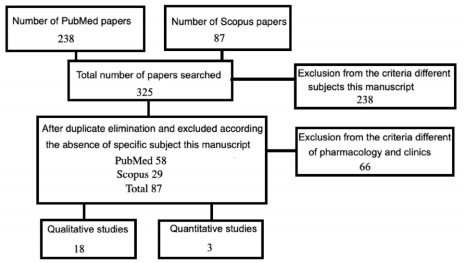
Flowchart of papers selected for this review following the PRISMA method. For more details about the exclusion criteria, see the text.

Articles that did not focus on CBD pharmacology were excluded. Among similar papers, we chose the most recent; for instance, three papers presented the same title - *Corticobasal degeneration* -, as their content was similar, the newest one was selected. After this assessment, 58 papers remained from PubMed and 29 from Scopus (Figure 1).

Thus, 87 studies were analyzed based on the title words ‘pharmacology’ and ‘clinical trials,’ ‘diagnosis,’ ‘treatment,’ and ‘interventions,’ that is, underlying tauopathy and symptomatic management were included in both original and review articles. Regarding pharmacology, only 6 papers were chosen since the drugs suggested for CBD treatment were identical in the texts; 15 studies about clinical trials were selected, totaling 21 works. Among those 21 articles, 17 were retrieved from both PubMed and Scopus, one from Scopus only, and three exclusively from PubMed (Figure 1).

A description of each study was used to integrate principles of the approaches currently available for CBD treatment.

## RESULTS

The articles found about CBD revealed that treatments can belong to one of two main approaches: (1) research studies aimed at underlying tauopathy and (2) symptomatic management. The articles were categorized (based on CBD expert groups) into case reports, case series, and pilot clinical trials. The last category involves case reports, case series, and pilot clinical trials.

### Research studies aimed at underlying tauopathy

An open pilot clinical trial used lithium, a GSK-3 inhibitor, to treat 17 patients with progressive supranuclear palsy and CBD (ClinicalTrials.gov identifier NCT00703677) and was early interrupted due to severe side effects Moretti et al.^8^

A 12-week randomized, double-blind, placebo-controlled pilot trial of Davunetide (NCT01056965) in predicted tauopathies (12 patients with CBS, progressive supranuclear palsy, progressive non-fluent aphasia, and frontotemporal dementia with parkinsonism linked to chromosome 17) was conducted in 2010. It was recently interrupted due to the lack of benefits.^2^

### Symptomatic management

A study using levodopa reported some improvement in 24% of patients.^5^ Other similar studies did not find a high level of benefits.^6^ Levodopa-induced dyskinesia can occur even in the absence of clinical benefits.^7^ Other dopaminergic drugs, such as dopamine agonists and selegiline, tend to produce less clinical improvement and more side effects than levodopa.^5^

Kompoliti et al.^5^ reviewed the medical records of 147 CBD patients from eight different medical centers, with only seven autopsy-proven cases in the sample. Parkinsonian features were present in all individuals, while other movement disorders were found in 89%, and cortical dysfunction in 93%. The most common parkinsonian signs were rigidity (92%), followed by bradykinesia (80%), gait disorder (80%), and tremor (55%). Other movement disorders identified were dystonia (71%), myoclonus (55%), apraxia (82%), and alien hand (42%), besides other features such as cortical sensory deficits (33%), and dementia (25%). Ninety-two percent of patients received dopaminergic drugs, which resulted in a beneficial effect for only 24%. Parkinsonian signs showed more improvement than other symptoms, and levodopa was the most effective drug. Benzodiazepines, mainly clonazepam, were administered to 47 patients, which improved myoclonus in 23% of them and dystonia in 9%. The most common disabling side effects in the clinical setting were somnolence and gastrointestinal complaints.

Baclofen, isolated or combined with an anticholinergic, reduced rigidity, but produced side effects.^5^ ^,^ ^6^ Clonazepam was beneficial for myoclonus and tremor.^5^ Many other drugs, including dopaminergic agonists, benzodiazepines, anticholinergics, propranolol, dantrolene sodium, and anticonvulsants, have been tested, usually without benefits and with potential side effects such as worse cognition.^5^ Botulinum toxin injections in the muscles of dystonic limbs provided symptomatic pain relief and prevented skin damage, particularly for wrist dystonia.^5^ ^,^ ^6^

Moretti et al.^8^ treated 51 patients with atypical parkinsonism (only 10 patients presented CBD) using rotigotine transdermal (a non-ergot dopamine agonist). The treated patients reported an overall decrease in tremor scores, with no increase in behavioral disturbances. The main side effects were hypotension, nausea, vomiting, somnolence, tachycardia, and dystonia. The authors suggested that rotigotine transdermal is effective and presents good tolerability in cases of atypical parkinsonism.

Kovács et et al.^9^ investigated the effects of levetiracetam (a new antiepileptic drug with antimyoclonic action) on myoclonus in two patients with CBD. The drug significantly decreased myoclonic activity in both patients at a dose of 1,500 mg/day. Cho et al.^10^ reported that a 72-year-old woman with probable clinical CBD and spontaneous rhythmic myoclonus in the right foot experienced symptom improvement with levetiracetam treatment. The effects of levetiracetam were associated with decreased amplitude of cortical somatosensory evoked potentials, which is increased in CBD. The authors indicated that the antimyoclonic effects of levetiracetam could be mediated by suppression of increased cortical excitability.

The suggested use of serotonin reuptake inhibitors for depression treatment in CBD is based only on the clinical experiences of a few authors,^11^ ^,^ ^12^ as is also the case with symptoms of obsessiveness and anxiety.^12^

Attempts to treat apathy have included acetylcholinesterase inhibitors (AChEIs) and psychostimulants,^12^ yet it remains difficult to treat. Apathy can worsen with selective serotonin reuptake inhibitors (SSRIs), and thus monitoring is necessary. Atypical antipsychotics or mood stabilizers have been used for problematic and inadequate treatments.^12^

AChEIs have been used for CBD, based on the anecdotal experience,^12^ ^,^ ^13^ but their therapeutic potential for this condition remains unknown. The benefits of using memantine for CBD are uncertain as the subject has been scarcely studied. Psychostimulants have been cited in some accounts of CBD treatment but without proven clinical efficacy.^12^

Shehata et al.^14^ investigated low-frequency repetitive transcranial magnetic stimulation (rTMS) as a therapeutic tool for CBD. Twenty-six clinically diagnosed CBD patients (according to Cambridge criteria) were followed for 12-18 months while receiving low-frequency rTMS associated with pharmacological treatment and botulinum toxin injections. The unified Parkinson’s disease rating scale (UPDRS) and quality of life improved after three months of therapeutic intervention (p<0.001 and p<0.05, respectively). No significant deterioration of cognitive function was detected during the study period. Caregiver time burden significantly decreased three months after treatment (p<0.01), which was maintained until 18 months. The authors concluded that CBD patients could benefit from this multidisciplinary therapeutic approach using low-frequency rTMS.

## DISCUSSION

Currently, atypical parkinsonian disorders such as CBD have no effective treatments approved by the United States Food and Drug Administration (FDA).^15^ The low efficacy of the drugs currently available is probably related to the wide distribution of pathological changes that explain the varied and complex spectrum of CBD clinical manifestations.^16^ Recent neuropathology and physiopathology discoveries have shed new light on CBD, but modifying therapies for this disease have yet to be found. The strategies of available treatments are based on a few clinical trials, case series, and mainly case reports.

Given that CBD is correctly diagnosed before death only in 25-56% of cases,^2^ ^,^ ^16^ ^,^ ^17^ discussions about the limitations of safely testing modifying agents are pertinent. Therefore, CBD seems not to spark interest in a greater number of clinical trials due to its low prevalence and the low reliability of clinical diagnosis.^18^

Current CBD treatment remains symptomatic and based on data from other similar diseases, such as Parkinson’s disease, AD, and frontotemporal dementia, because of the lack of specific studies on CBD.^2^ ^,^ ^4^ The symptomatic treatment of CBD patients can sometimes help improve motor symptoms, but the effects are usually unsatisfactory. Due to the lack of pharmacological alternatives currently available, there are no pharmacological strategies or palliative care for the multidisciplinary integration of therapeutic components for CBD patients.^12^

Therapeutic agents for the symptomatic treatment of Parkinson’s disease (levodopa or dopamine agonists) are used for the management of motor symptoms in CBD (Table 1). Moretti et al.^8^ suggested that rotigotine transdermal is effective for treating atypical parkinsonism, but did not define its efficacy for each subtype of parkinsonism. Furthermore, they provide no evidence that rotigotine is more effective in treating CBD than other dopaminergic agonists. The main limitation to using dopaminergic agents is the high likelihood of inducing psychotic adverse events and other severe psychiatric symptoms, such as hypersexuality, compulsive shopping, pathological gambling,^19^ dyskinesias, palpitations, nausea, depression, and urinary retention.^20^

**Table 1.** Actions and side effects of the main drugs cited in this work.

Drug	Actions	Side effects
Lithium	Mood stabilizer. Reduced gambling thoughts and behaviors in bipolar disorders^19^	Confusion, somnolence
Serotonin reuptake inhibitors	Decreased pursuit of rewards, hypersexuality, and aggression,^20^ improved social and occupational functioning^19^	Dry mouth, diarrhea, nausea, loss of appetite, headache, somnolence, insomnia, tremor, agitation, sexual dysfunction, asthenia, dizziness, anxiety, nervousness^2^ ^,^ ^20^
Levodopa	Dopamine agonist^19^	Dyskinesia, cardiac irregularities, orthostatic hypotensive episodes, psychotic episodes, nausea, depression, urinary retention^20^
Rotigotine	Non-ergot dopamine agonist^8^	Hypotension, nausea, vomiting, somnolence, tachycardia, and dystonia^8^
AChEIs	Acetylcholinesterase inhibitors^12^ ^,^ ^13^	Nausea, vomiting, diarrhea, anorexia, insomnia, fatigue, loss of appetite, weight loss, abdominal pain^2^ ^,^ ^20^
Clonazepam	Hypnotic and anxiolytic	Drowsiness,^2^ ^,^ ^4^ ^,^ ^21^ behavioral changes, imbalance, incoordination, dizziness,^2^ ^,^ ^21^ tiredness, confusion, memory loss,^4^ somnolence, gastrointestinal complaints^5^, slurred speech, cognitive impairment, depression^21^
Levetiracetam	Decreased amplitude of cortical somatosensory evoked potentials^8^	Behavioral changes, irritability, fatigue, loss of appetite, dizziness, headache^2^
Botulinum toxin	Acetylcholine antagonist	Excessive weakness^2^

Despite the benefits observed in some patients, there is no indication that levetiracetam could be more effective than other myoclonic agents in CBD treatment. On the other hand, benzodiazepines, clonazepam, for instance, improved myoclonus and dystonia^5^ and seem to be one of the best options.^21^

Therapeutic agents approved for AD, such as AChEIs and memantine, have been used off-label to treat cognitive and behavioral symptoms in tauopathies, but results have not been consistent.^13^ Thinking that some CBS and CBD presentation phenotypes might respond to AChEIs depending on the associated pathology may be tempting: for example, patients with clinical presentation of CBS and AD. This would explain why some patients respond to these drugs and others do not.^2^ Given the current uncertainty regarding the proper identification of pathological diagnosis in patients with CBS and other phenotypes associated with CBD, trying AChEIs may be reasonable since patients with certain underlying pathologies, such as AD, may experience some improvement.

However, AChEI side effects, such as nausea, insomnia, fatigue, vomiting, anorexia, diarrhea, weight loss, and abdominal pain, must be taken into account.^5^ ^,^ ^20^ In the same way, memantine side effects, including insomnia, confusion, dizziness, headache, agitation, and hallucination, can result in discontinuation.^20^

Despite the lack of formal clinical trials aimed at psychiatric symptoms, such as depression and anxiety, in patients with CBD, treating associated behavioral manifestations is essential for alleviating symptoms that have effective pharmacological interventions and thus improve the quality of life of patients. New antidepressants, such as vortioxetine, may have a better pharmacological profile for treating depressive symptoms and executive dysfunction in CBD and need to be tested, regardless of the pharmaceutical industry’s apparent lack of interest in CBD, as suggested by the scarcity of recent clinical trials with new drugs.

After rationally compiling data from the cited literature, pharmacological interventions must be adjusted for the specific symptoms of each patient, and decisions about the time of treatment must be based on its efficacy for each individual according to their tolerances.^20^ ^,^ ^21^

CBD seems not to spark interest in more clinical trials due to its low prevalence and the low reliability of clinical diagnosis. Most pharmacological agents used for CBD were indicated based on data from correlated diseases or functional primary psychiatric syndromes (depression, anxiety), while a specific therapy to tackle CBD symptoms remains non-existent.

Symptomatic treatment of CBD patients could be useful for improving motor symptoms (parkinsonism, dystonia, myoclonus), but the effects are generally unsatisfactory. In order to handle the behavioral manifestations associated with CBD, we must treat the symptoms that have effective pharmacological interventions, aiming at improving the quality of life of patients.
